# Erasing the Past: A New Identity for the Damoclean Pathogen Causing South American Leaf Blight of Rubber

**DOI:** 10.1371/journal.pone.0104750

**Published:** 2014-08-15

**Authors:** Braz Tavares da Hora Júnior, Davi Mesquita de Macedo, Robert Weingart Barreto, Harry C. Evans, Carlos Raimundo Reis Mattos, Luiz Antonio Maffia, Eduardo S. G. Mizubuti

**Affiliations:** 1 Departamento de Fitopatologia, Universidade Federal de Viçosa, Viçosa, Minas Gerais, Brazil; 2 CAB International, E-UK Centre, Egham, Surrey, United Kingdom; 3 Plantações Michelin da Bahia, Ituberá, Bahia, Brazil; Portland State University, United States of America

## Abstract

**Background:**

South American leaf blight (SALB) of rubber has been the main constraint to production in its neotropical centre of origin since commercial plantations were first established. The fungal causal agent was identified and described more than a century ago but its precise placement within the Ascomycota still remains uncertain. Indeed, such is the ambiguity surrounding the pathogen that each of the spore morphs would, according to their present classification, be placed in different ascomycete families: the *Microcyclus* sexual morph in the Planistromellaceae and the two purported asexual morphs - *Fusicladium* and *Aposphaeria* – in the Venturiaceae and Lophiostomataceae, respectively. Given the historical importance of the fungus and the ever-menacing threat that it poses to rubber production in the Palaeotropics – and, thus to the rubber industry and to the global economy – its phylogeny, as well as its biology, should be resolved as a matter of urgency.

**Methods and Results:**

Here, six genomic regions (LSU rRNA, mtSSU, MCM7, EF-1α, Act and ITS) were used for reconstructing the molecular phylogeny of the SALB fungus based on material collected throughout Brazil. The analyses support the classification of the fungus in the family Mycosphaerellaceae s. str. (Capnodiales, Dothideomycetes) and place it firmly within the clade *Pseudocercospora* s. str., now accepted as one of the distinct genera within Mycosphaerellaceae. The new combination *Pseudocercospora ulei* is proposed and the life cycle of the fungus is confirmed, based on both experimental and phylogenetic evidence, with the *Aposphaeria* morph shown to have a spermatial rather than an infective-dispersal function.

**Conclusions:**

Because the phylogeny of the SALB fungus has now been clarified, new insights of its epidemiology and genomics can be gained following comparison with closely-related, better-researched crop pathogens.

## Introduction

South American leaf blight (SALB) of the rubber tree *Hevea brasiliensis* (Willd. ex A. L. Juss.) Muell.-Arg., caused by *Microcyclus ulei* (Henn.) Arx (Ascomycota), is recognized as the most serious threat to the natural rubber industry worldwide [1]–[4]. Epidemics of SALB led to the failure of rubber plantations in tropical America in the early 20^th^ century, as epitomized by the demise of Fordlandia in the Lower Amazon region of Brazil despite enormous investment in research and development [5], [6]. Currently, the world supply of natural rubber is highly dependent on the plantations established in Southeast Asia [3]. The magnitude of the threat represented by the SALB fungus is highlighted by Money [7] who described the relevance of natural rubber as an irreplaceable prime matter for the world's industry in a plethora of applications besides tires, machinery belts and condoms and as an industry itself providing the livelihood of 30 million people, concluding that “Nothing else (but *M. ulei*) has the power to terminate the global flow of latex.” Because of the potential serious economic consequences, there are strict quarantine measures in place to prevent SALB from establishing in the rubber tree production areas in the Palaeotropics, especially in Southeast Asia, a SALB-free zone [3], [8]. The fungus infects young leaves, stems and fruits of *Hevea brasiliensis*, as well as *H. benthamiana* Muell.-Arg., *H. spruceana* (Benth.) Muell.-Arg., *H. guianensis* Aublet and *H. camporum* Ducke [2], resulting in defoliation and, potentially, after repeated outbreaks in tree death.

The fungus was first observed and collected by E. Ule in 1900 in the Upper Amazon region of Peru and Brazil and was later described by Hennings [9]. Initially, two spore morphs were recognised: the sexual morph, *Dothidella ulei*; and a supposed asexual pycnidial morph, *Aposphaeria ulei*. The hyphomycete asexual morph was described soon after by J. Kuyper in Surinam in 1911 as *Fusicladium macrosporum*. In 1917, G. Stahel observed the connection of hyphae from different fungal structures within the leaf tissue and linked the sexual and asexual morphs of the fungus and renamed the former as *Melanopsammopsis ulei* [1]. Much later, von Arx (in [10]) transferred this to the genus *Microcyclus* and suggested a close relationship with the genus *Mycosphaerella*, based on the morphology of the hyphomycete *Passalora*-type morph. Subsequently, he suggested that *Fusicladium* should only be used for asexual morphs belonging to the family Venturiaceae rather than to the Mycosphaerellaceae [11]. *Microcyclus* is characterized by erumpent ascostromata on living leaves having a foot-like hypostroma, similar to some *Mycosphaerella* pathogens of pine trees [12].

Each of the spore morphs in *M. ulei*'s life cycle would, according to the present classification for the genera where they are placed, belong to a different ascomycete family, namely: Planistromellaceae/incertae sedis (*Microcyclus* sexual morph) [13]–[15], Venturiaceae (*Fusicladium* asexual morph) [16] and Lophiostomataceae (*Aposphaeria* asexual morph) [17], [18]. This is clearly inadequate and requires an explanation. Although there would be grounds for speculating that SALB is a disease complex involving three unrelated fungal species, perhaps with the involvement of a mycoparasite, previous authors that have dealt with the SALB disease and its etiology have not reached such conclusion Nevertheless, Langford [19] and more recently, Guyot and Doaré [20] have inoculated conidia and ascospores on rubber plants and were able to reproduce the symptoms of SALB, demonstrating that the *Microcyclus* and *Fusicladium* morphs are part of the cycle of a single fungus. Ascospores were shown to play an essential role in the perpetuation of the disease outside the host's growth periods, in the resumption of epidemics, and in long-distance dispersal and the conidia contributed primarily to the stepwise and short-distance spread of the disease [21]. A conclusive Koch's postulates have never been performed with the pycnidial morph of the fungus. This might play a different role in the life cycle of the fungus or even be a mycoparasite of *M. ulei*. Conversely, this puzzling situation may just result from the lack of proper understanding of the life cycle and classification of the fungus behind SALB.

The general lack of DNA sequence data for all three purported morphs (*Microcyclus*, *Fusicladium* and *Aposphaeria*) contributes to the confusion surrounding the taxonomy of the causal agent of SALB. Until relatively recently, the genus *Microcyclus* was classified in the Mycosphaerellaceae (order Capnodiales), as a stromatic counterpart of the family [22], [23], but has since been re-classified in the Planistromellaceae (Dothideales), initially to accommodate genera with ascostromatal locules that open schizogenously by a periphysate ostiole [13], [14]. More recently, a phylogenetic analysis showed that the core Planistromellaceae belong in the Botryosphaeriales, from which *Microcyclus* – represented only by ITS sequences of *M. ulei* – was excluded based on BLAST searches of GenBank, and its familial position was considered to be uncertain [15]. After a taxonomic review of the hyphomycete conidial morph, this asexual morph was retained in *Fusicladium* s. lat. [16]; some species of which have now been assigned to the newly recognised family Sympoventuriaceae in the new order Venturiales [24]. However, in the absence of type material, the species was neotypified and the name changed to *F. heveae* since it was adjudged that the original epithet could be confused with *F. macrosporium* Bonord. 1864 [16], [25]. The latter authors added the rider that: “*Fusicladium heveae* is an unusual species, since its teleomorph, *Microcyclus ulei*, is placed in the Mycosphaerellaceae and not in the Venturiaceae”. The coelomycete genus *Aposphaeria* is recognized as a member of the family Lophiostomataceae (order Pleosporales), as a well-supported group [17], [18]. Thus, questionable issues regarding the classification of both the purported asexual morphs, as well as the sexual morph, at the genus, family and order levels of the causal agent of SALB need to be addressed. “Clearly, a re-examination of its taxonomic position would be justified” [26] and a single unifying generic name should be adopted in accordance with the new nomenclatural rules of one fungus one name system and the promotion of progressive plant pathology [27].

Additionally, knowledge about the evolutionary history of *M. ulei* and of related species is scarce and molecular studies could help to resolve the true affinity of this fungus [15], [25], [28]. Thus, the objectives of the present study were: i) To obtain molecular evidence of the connection of the three spore morphs of *M. ulei*; ii) to elucidate the phylogenetic relationships of *M. ulei* using molecular approaches; iii) to determine the adequate nomenclatural treatment for the fungus causing SALB; iiii) to obtain experimental evidence on the function of the intermediate pycnidial morph; iv) to prepare an updated model of life-cycle of the SALB fungus. Conceivably, this should also lead to a better understanding of the biology and ecology of one of the most threatening plant pathogens to mankind's welfare.

## Material and Methods

### Ethics statement

No specific permits were required for the described field studies. No endangered or protected species were involved in the studies.

### Sampling, isolation and DNA extraction

Leaves with lesions of South American leaf blight were sampled in commercial fields of rubber in Brazil. Sampling was aimed at areas with records of high incidence of SALB in the Brazilian states of Acre, Rondônia, Mato Grosso, Minas Gerais, Espírito Santo and Bahia between 2008 and 2010 (Table 1). Single conidia were transferred from fungal structures formed on lesions to culture media, using a sterilized fine-needle under a dissecting microscope. Monosporic cultures of *F. heveae* were grown on M4 culture medium [29] in the dark for 2 months at 24 ± 1 °C. Pycnidial stromata of *A. ulei* and ascostromata of *M. ulei* were excised from a single lesion of an infected leaf with a sterilized razor blade. Each lesion was examined under the microscope to check for possible contamination by mycoparasites and selected stromata (approximately 10 structures) were transferred to a microtube (1.5 mL). The procedure was repeated from another lesion on the same leaf. To break up the melanised cell walls, the microtubes containing fungal material (mycelium, pycnidia or ascostromata) were placed in liquid nitrogen and macerated using a micropestle. DNA extraction was carried out following standard cetyltrimethyl ammonium bromide extraction procedures [30].

**Table 1:** Origin of the *Microcyclus ulei* isolates used in the phylogenetic study.

Isolate	Location^1^	Coordinates in decimals (Lat/Lon)	GenBank accession number (ITS, ACT, EF-1α, LSU, MCM7, mtSSU)
*Fusicladium heveae* UFVMu01RO	Buritis-RO	-10.211944/-63.828889	KC800717, KC800725, KC800733, KC800741, KC800755, KC800768
*Fusicladium heveae* UFVMu05MT	Itiquira-MT	-17.208889/-54.150000	KC800718, KC800726, KC800734, KC800742, KC800756, KC800769
*Fusicladium heveae* UFVMu01ES	Sooretama-ES	-19.220087/-40.121414	KC800719, KC800727, KC800735, KC800743, KC800757, KC800770
*Fusicladium heveae* UFVMu77BA	Porto Seguro-BA	-16.378001/-39.366433	KC800720, KC800728, KC800736, KC800744, KC800758, KC800771
*Microcyclus ulei* AC	Xapuri-AC	-10.651944/-68.503889	KC800721, KC800729, KC800737, KC800745, KC800759, KC800772
*Microcyclus ulei* MG	Oratórios-MG	-20.415833/-42.908889	KC800722, KC800730, KC800738, KC800746, KC800760, KC800773
*Aposphaeria ulei* RO	Ariquemes-RO	-9.913333/-63.040833	KC800723, KC800731, KC800739, KC800747, KC800761, KC800774
*Aposphaeria ulei* ES	Cachoeiro do Itapemirim-ES	-20.752609/-41.290358	KC800724, KC800732, KC800740, KC800748, KC800762, KC800775

1 Brazilian states: Acre (AC), Bahia (BA), Espírito Santo (ES), Mato Grosso (MT), Minas Gerais (MG) and Rondônia (RO).

### DNA phylogeny

All phylogenetic analyses were performed using DNA sequence of six loci as the first 900 bp at the 5′ end of the 28S rRNA gene (LSU), the first and second internal transcribed spacer (ITS), the mitochondrial region of the mtSSU-rDNA and partial sequences of nuclear genes such as the mini-chromosome maintenance protein (MCM7), translation elongation factor 1-alpha (EF-1α) and actin (ACT). Specific primers utilized were LR0R [31] and LR5 [32], ITS1 and ITS4 [33], NMS1 and NMS2 [34], Mcm7-709for and Mcm7-1384rev [35], EF1-728F and EF1-986R and ACT-512F and ACT-783R [36], respectively.

The polymerase chain reaction (PCR) was done with a mixture containing 20 ηg of DNA, 0.2 µM of each primer and 1× of DreamTaq DNA Polymerase Master mix as described by the manufacturer (Thermo Fisher Scientific). PCR cycles were carried out in a PTC100 thermal cycler (MJ Research, Incline Village, NV) and consisted of a 5 min denaturation step at 94 °C, followed by 35 cycles of 30 s at 94 °C, 30 s at 60 °C for LSU, mtSSU, EF-1α, ACT and ITS primers or 57 °C for MCM7 primers and 1 min at 72 °C with a final extension of 10 min at 72 °C. PCR products were visualized by ultraviolet fluorescence following 1% agarose gel electrophoresis in 1× TBE buffer and GelRed (Biotium) staining. Single-band products were purified using the E.Z.N.A cycle-pure kit (OMEGA Bio-tek). DNA concentration was measured by NanoDrop 2000 Spectrophotometer (Thermo Fisher Scientific). The same primers used for PCR amplification were used for the sequencing reactions using the DYEnamic ET Terminator Cycle Sequencing Kit (GE Healthcare) according to the manufacturer's recommendations. The purified PCR products were sequenced using a MegaBACE 1000 DNA Sequencing System (GE Healthcare). A consensus sequence was generated after manually editing with The Staden Package, v. 1.6.0 [37]. Genbank accession numbers are provided in Table 1. Additional sequences used in the analyses were obtained from GenBank and the Fungal Genomics Portal of the Joint Genome Institute [38](Table S1). Sequences were aligned with the Muscle v. 3.6 software [39] implemented in the MEGA 5.0 program [40]. Statistics resulting from sequence alignment such as variable, parsimony-informative and uninformative sites were estimated in MEGA.

Bayesian analysis was conducted with MrBayes v. 3.1.2 [41] to determine generic relationships based on the LSU, mtSSU and MCM7. Aligned datasets were inspected with MrModeltest v.2.2 [42] to select the suitable nucleotide substitution model and all trees were rooted with *Aspergillus niger*. Additionally, another dataset at species level was constructed and Bayesian phylogeny was derived from the concatenated ITS, EF-1α and ACT alignments with *Pseudocercospora* s. str. sequences. *Passalora eucalypti* was used as the outgroup. For this analysis, the alignment gaps were treated as a fifth character state and the MrModeltest v. 2.2 selected the best nucleotide substitution model for each partition. The Markov Chain Monte Carlo (MCMC) analysis used four chains that started with a heating parameter of 0.2 from a random tree topology and lasted 50 million generations. Trees were saved each 1000 generations, resulting in 50,000 saved trees. Burn-in was set at 5,000,000 generations after which the likelihood values were stationary, leaving 35,000 trees from which the 50% majority rule consensus trees and posterior probabilities were calculated. Quality of mixing and convergence to the stationary distribution were assessed from three independent runs using Tracer v. 1.5 [43]. The resulting phylogenetic trees were prepared using FigTree v. 1.4 (http://tree.bio.ed.ac.uk/software/figtree). All alignments and resulting trees were deposited into TreeBASE (14357), and the nomenclatural novelty in MycoBank [44].

### Taxonomy

Based on newly obtained information and information available in the literature and on the re-examination of newly collected material a model was prepared. Observations of the morphology of fungal structures belonging to each morph in the life cycle were made based on the examination of microscope slides containing sections of such structures mounted in lactophenol or lactofuchsin and observed under a light microscope (Olympus BX 51) equipped with a drawing tube. At least 30 measurements were made of each fungal structure.

### Nomenclature

The electronic version of this article in Portable Document Format (PDF) in a work with an ISSN or ISBN will represent a published work according to the International Code of Nomenclature for algae, fungi, and plants, and hence the new names contained in the electronic publication of a PLOS ONE article are effectively published under that Code from the electronic edition alone, so there is no longer any need to provide printed copies.

In addition, the new combination introduced in this work has been submitted to MycoBank from where they will be made available to the Global Names Index. The unique MycoBank number can be resolved and the associated information viewed through any standard web browser by appending the MycoBank number contained in this publication to the prefix http://www.mycobank.org/MB/. The online version of this work is archived and available from the following digital repositories: [PubMed Central, LOCKSS].

### Assessments of the pleomorphic development of *Microcyclus ulei* under natural conditions

The development of the pathogen in the rubber leaf was monitored under environmental conditions favorable to the development of SALB. At the Michelin Plantation of Bahia (Brazil), 90 leaves at the B2 developmental stage [45] of eight rubber trees of the RO38 clone were tagged with a label and observations of the disease were made until maturity (stage D), from December 15, 2011 to February 24, 2012 (Experiment 1) and September 19 to December 03, 2012 (Experiment 2). All trees were pruned 45 days before each experiment started. Scoring of sporulation in lesions naturally infected was performed at every four days using a 1–6 scale for sporulation intensity of the asexual morph (conidia) adapted from Junqueira et al. [46], where 1  =  necrotic non-sporulating lesions, 2  =  chlorotic non-sporulating lesions, 3  =  slight sporulation on lower side of the leaflets, 4  =  moderate sporulation on lower side of the leaflets, 5  =  high sporulation on lower side of the leaflets, and 6  =  high sporulation on both sides of the leaflets. Pycnidial and ascostromata density was assessed at the same time interval using a 0–4 scale where 0  =  no stroma, 1  =  1–5 stromata per leaflet, 2  =  6–15 stromata per leaflet, 3  =  16–50 stromata per leaflet, and 4  =  more than 50 stromata per leaflet. The weighted average was computed in each observation from total of leaves in each phenological stage and score of conidial sporulation intensity and spermogonia and ascostromata density.

### Test of infectivity and germination of pycniospores of *Microcyclus ulei* under controlled conditions

Suspension of pycniospores was obtained from pycnidia formed in near mature leaves (C/D stage) of the RO38 rubber clone. There were no conidia or ascospores. Suspension of hyphomycete asexual morph was used as positive control. Both inoculum suspensions were adjusted to 2×10^5^ spores/mL in a Tween 80 at 0.05% solution. The lower surface of three young leaves from the Fx 3864 rubber tree clone were spray-inoculated until runoff with an inoculum suspension of pycniospores or conidia separately using a HS Airbrush Complete set (Paasche Airbrush company) in an inoculation chamber at 24°C, relative humidity greater than 85%, artificial daylight of 2000 lux and 12 h photoperiod. The 0.05% Tween 80 solution was used as a negative control. Sporulation was scored after 12 days on all inoculated leaves. The suspensions of pycniospores and conidia were incubated in the dark at 25 °C on both water agar and M4 culture media. Germination assessments were conducted at 6, 12, 24 and 120 h of incubation at 24 ±1°C. The experiment was conducted twice.

## Results

### Phylogeny: LSU, mtSSU and MCM7 datasets

**Figure 1:**
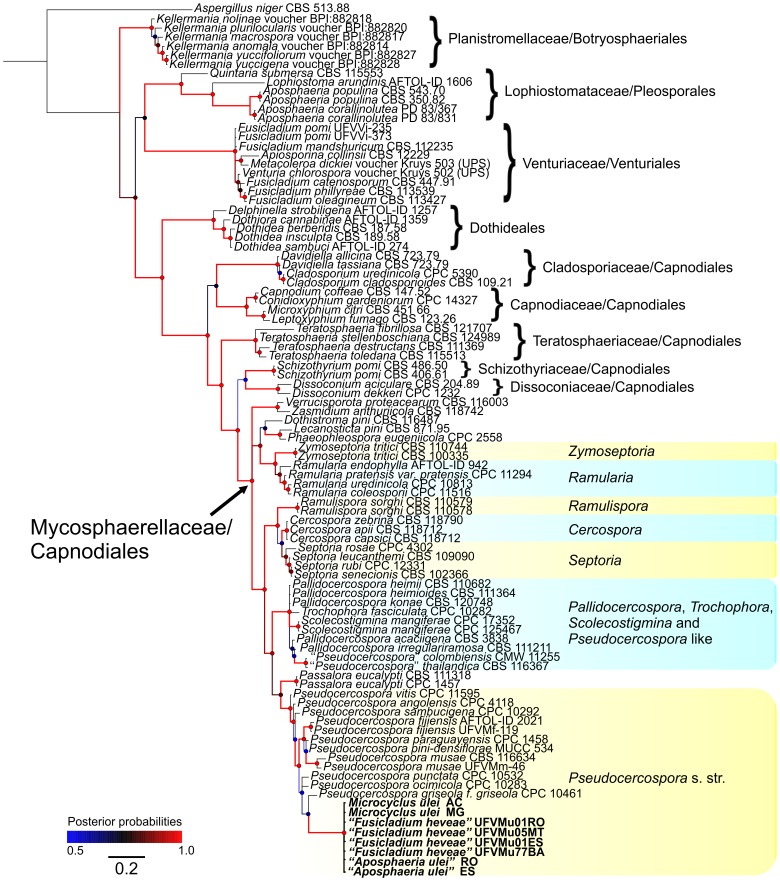
Bayesian analysis showing the phylogenetic relationships of *Microcyclus ulei* based on the LSU sequence alignment. Bayesian posterior probabilities are given at the nodes and coded according to the colored scale bar. The black line scale bar shows 0.2 expected changes per site. The tree was rooted with *Aspergillus niger*.

**Figure 2:**
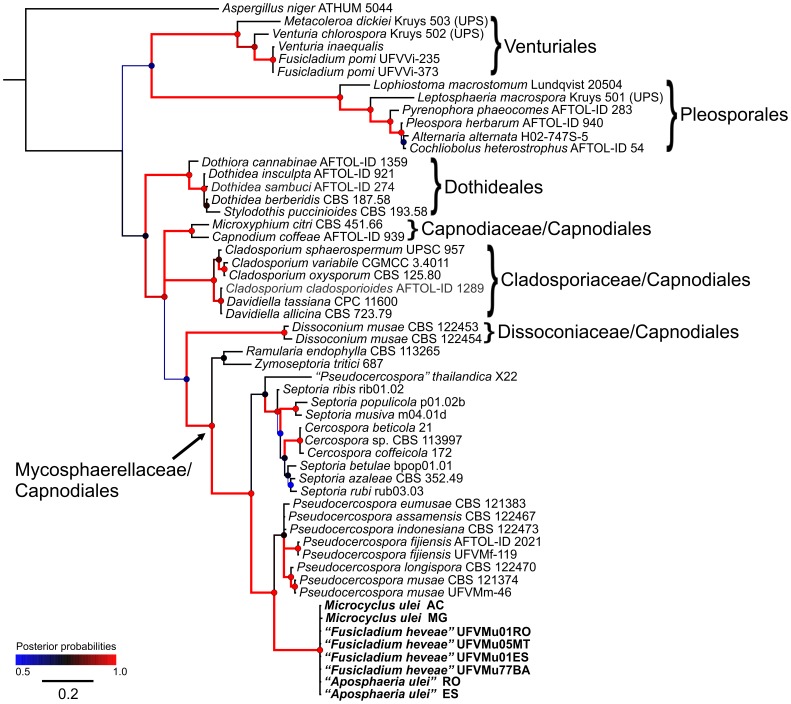
Bayesian analysis showing the phylogenetic relationships of *Microcyclus ulei* based on the mtSSU sequence alignment. Bayesian posterior probabilities are given at the nodes and coded according to the colored scale bar. The black line scale bar shows 0.2 expected changes per site. The tree was rooted with *Aspergillus niger*.

**Figure 3:**
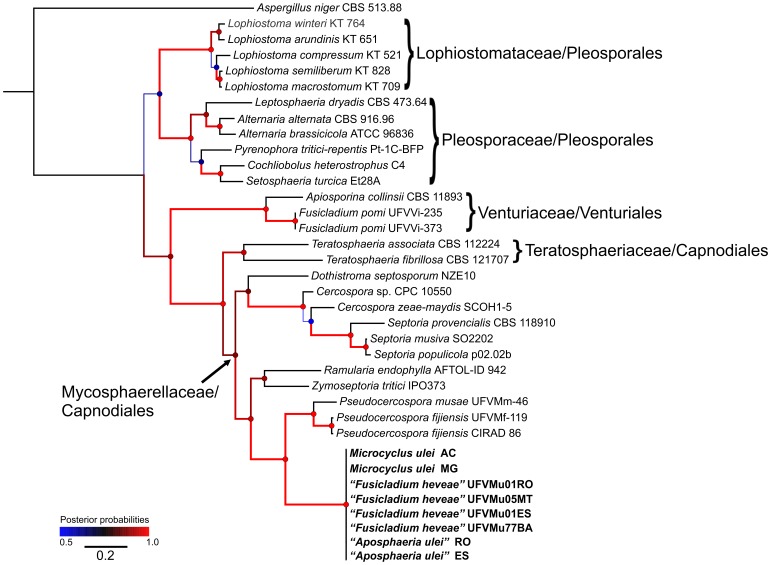
Bayesian analysis showing the phylogenetic relationships of *Microcyclus ulei* based on the MCM7 sequence alignment. Bayesian posterior probabilities are given at the nodes and coded according to the colored scale bar. The black line scale bar shows 0.2 expected changes per site. The tree was rooted with *Aspergillus niger*.

Strongly supported clades provide molecular evidence of asexual-sexual morph connection between the three morphs of the SALB fungus and thus the holomorph belongs to the family Mycosphaerellaceae s. str., order Capnodiales (Figures 1–3). For this study, two specimens each of *M. ulei* and *A. ulei* and four of *F. heveae* collected in Brazil were analyzed. The generic relationships were determined with datasets for LSU, mtSSU and MCM7 that included 89, 55 and 36 taxa, respectively (available in TreeBASE) and the same nucleotide substitution model, GTR+I+G was used in all analyses.

The alignment of the partial sequence of the LSU region had 838 sites including alignment gaps, of which 243 sites were parsimony-informative, 57 were variable and parsimony-uninformative, and 534 were constant. The LSU phylogeny (Figure 1) resulted in *Aposphaeria populina* and *A. corallinolutea* (members of the Lophiostomataceae: Pleosporales), species of the genus *Fusicladium* (members of the Sympoventuriaceae: Venturiales), species of *Kellermania* (members of Planistromellaceae: Botryosphaeriales), as well as members of the Dothideales forming well-supported monophyletic groups. Representatives of the Capnodiales grouped within well-established families as Cladosporiaceae, Capnodiaceae, Teratosphaeriaceae, Schizothyriaceae, Dissoconiaceae and Mycosphaerellaceae. In the Mycosphaerellaceae, several well-supported clades were formed with *Mycosphaerella* s. str. (asexual morph *Ramularia*) and mycosphaerella-like with the asexual morphs *Cercospora*, *Pallidocercospora*, *Pseudocercospora*, pseudocercospora-like, *Ramulispora*, *Septoria*, and *Zymoseptoria*, amongst others. *Microcyclus ulei* and its morphs *A. ulei* and *F. heveae* were identical and grouped in the well-defined *Pseudocercospora* s. str. clade of the Mycosphaerellaceae, distinct from *Mycosphaerella* s. str. (*M. punctiformis*, represented by *Ramularia endophylla*), showing clearly that the holomorph of the SALB fungus is a species of *Pseudocercospora* in the Mycosphaerellaceae.

The phylogeny reconstructed with the partial sequence of the mtSSU sequences (Figure 2) had 724 characters (248 parsimony-informative and 99 singletons), while the dataset of the partial sequence of the MCM7 region (Figure 3) was based on a dataset with 466 characters (254 variables sites of which 221 were parsimony-informative). The OTUs from the Venturiales, Pleosporales, Dothideales and Capnodiales (Capnodiaceae, Cladosporiaceae, Dissoconiaceae and Mycosphaerellaceae) for mtSSU region, and those from the Pleosporales (Lophiostomataceae and Pleosporaceae), Venturiales and Capnodiales (Teratosphaeriaceae and Mycosphaerellaceae) for MCM7 formed well-supported clades. In both analyses, OTUs of the genus *Pseudocercospora* in Mycosphaerellaceae were the nearest relatives of the holomorph of the SALB pathogen.

### Phylogeny: Concatenated ITS, EF-1α and ACT datasets

**Figure 4:**
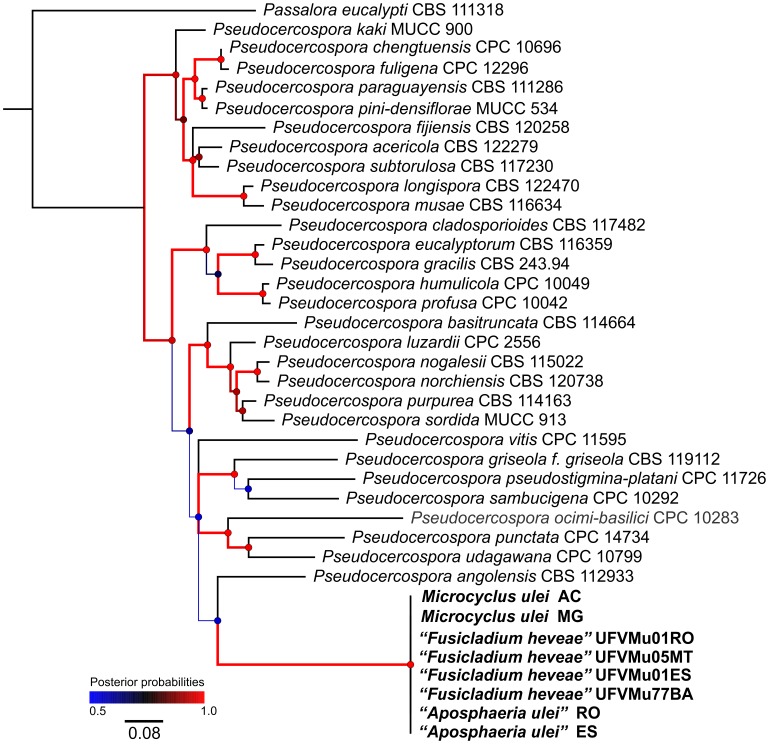
Phylogenetic relationships of *Microcyclus ulei* based on the combined ITS, EF-1α and ACT sequences alignment. Bayesian posterior probabilities are given at the nodes and coded according to the colored scale bar. The black line scale bar shows 0.08 expected changes per site. The tree was rooted with *Passalora eucalypti*.

After the analyses at the genus level, phylogeny at species level was conducted with some OTUs of *Pseudocercospora* s. str. using sequences of ITS, EF-1α and ACT regions combined (Figure 4). The nucleotide substitution models, GTR+I+G, GTR+G and SYM+I+G, were used for each partition, respectively. For this dataset, 1126 characters were used, 517 were constant, 367 were parsimony-informative and 144 were singletons. Two well-defined clades were observed, both with posterior probability of 0.96, and the holomorph was closely related to *Pseudocercospora angolensis*.

### Pleomorphic development and function of intermediate pycnidial morph in the life cycle of *Microcyclus ulei*

**Figure 5:**
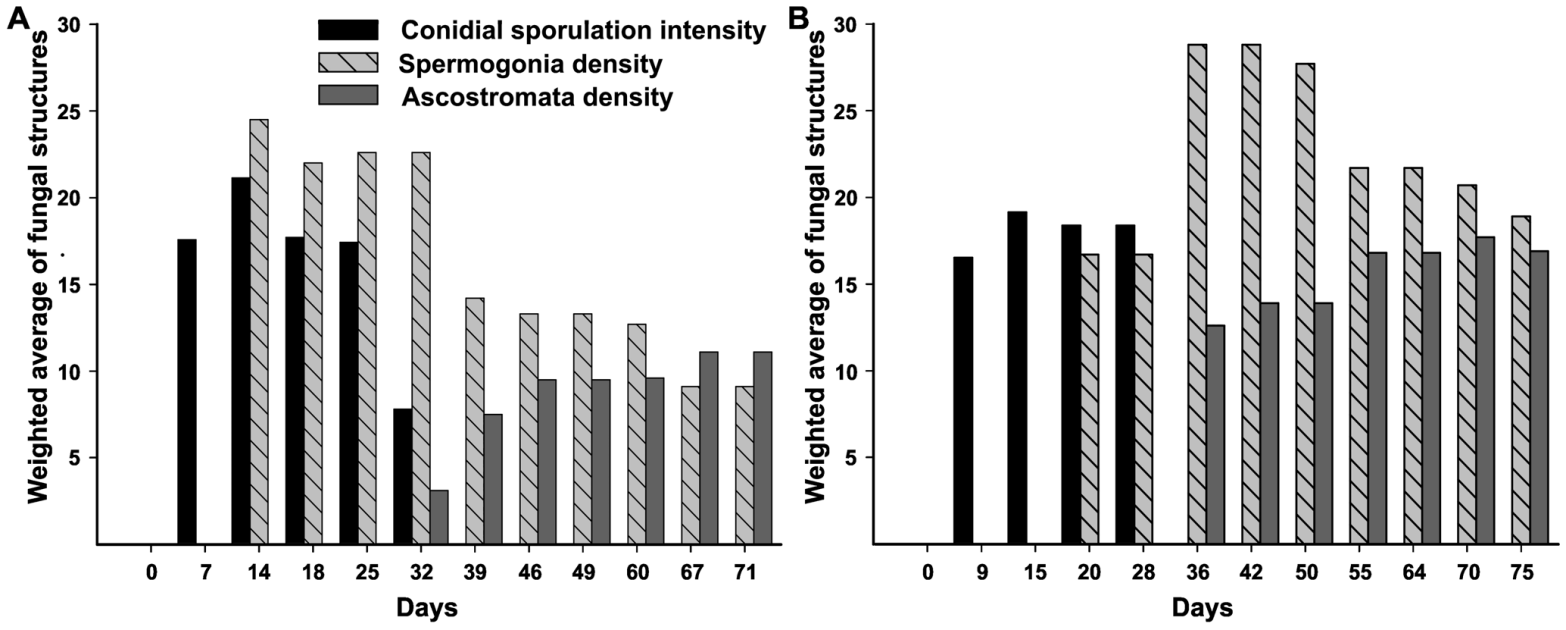
Pleomorphic development of the life cycle of *Pseudocercospora ulei*. Weighted average based on the score of conidial sporulation intensity and spermogonia and ascostromata density during the leaf development of RO38 rubber clone grown under field conditions. Assessments were made from December 15, 2011 to February 24, 2012 (A); and from September 19 to December 3, 2012 (B).

The SALB symptoms were assessed in two consecutive experiments from trees after pruning. In the first period, December 15, 2011 to February 24, 2012, conidial lesions started in leaves in the B2 stage on December 19 and were observed up to the D stage leaves, which corresponded to 26 days of monitoring (Figure 5A). *A. ulei* first emerged from the upper side of infected leaves in the C/D stage on December 29. Ascostromata arose after 32 days (January 17) and were found only in the upper side of D stage leaves. In the second period, September 19 to December 3, 2012, conidial lesions were found on September, 28 in B2 stage leaves and in D leaves within a 28 day-period (October 17) (Figure 5B). *A. ulei* appeared in the C/D stage on October 9 (20 days) and ascostromata arose after 36 days of monitoring and were found only in stage D leaves. Both stages occurred in the adaxial side of leaves.

The main weather descriptors during the course of the experiment 1 (72 days) and experiment 2 (75 days) were, respectively: average maximum temperature 29 and 27.7 °C; average minimum temperature 22 and 20.2 °C; average relative humidity 83% and 83.9%. Total (cumulative) rainfall was 267 and 267.8 mm.

The possible contribution of the so-called pycniospores for disease initiation was investigated by inoculating a concentrated suspension of “pynciospores” onto young leaflets under controlled conditions and also by assessing spore germination. Inoculation of pycniospores did not cause lesions, but signs of the disease were visible in leaflets inoculated with conidia after 12 days. The pycniospores did not germinate *in vitro*, on both culture media, while conidia germination started at 6 h of incubation.

### Taxonomy

Based on the multi-gene phylogeny analyses, the pleomorphic fungus *M. ulei* was shown to cluster unmistakably within the *Pseudocercospora* s. str. clade and, in accordance with Art. 59 of the ICN (International Code of Nomenclature for Algae, Fungi and Plants), a new combination is hereby introduced.

***Pseudocercospora ulei*** (Henn.) Hora Junior & Mizubuti, **comb. nov**. MB 804653 (Fig. 4)

Basionym: *Microcyclus ulei* (Henn.) Arx, in Müller & Arx, Beitr. Kryptogamenfl. Schweiz 11: 373 (1962).

≡ *Dothidella ulei* Henn., Hedwigia 43(4): 254 (1904).

≡ *Aposphaeria ulei* Henn. Notizbl. Bot. Gart. Berlin-Dahlem 4: 135 (1904)

≡*Apiosphaeria ulei* Henn., Hedwigia 43: 245 (1904)

≡ *Fusicladium macrosporum* J. Kuyper, Recueil Trav. Bot. Néerl. 8: 374 (1911).

 =  ?*Passalora heveae* Massee (nom. nud.) sensu Stahel, Bull. Dept. Landb. Suriname 34: 34 (1917).

≡ *Melanopsammopsis ulei* (Henn.) Stahel, Bull. Dep. Landb. Suriname 34: 1–111 (1917)

≡ *Fusicladium heveae* K. Schub. & U. Braun, in Crous & Braun, *Mycosphaerella* and its anamorphs: 1. Names published in *Cercospora* and *Passalora*. CBS Biodiversity Series 1: 481 (2003)

*Lesions* on young stems, petioles, inflorescences, fruits and (mainly) on leaves. *Sexual morph lesions*, initially punctiform, becoming circular to subcircular, necrotic, pale brown centrally surrounded with a ring of prominent black stromata, growing with age and leading to loss of subcircular fragments (shot-holes), 1–3 × 1–6 mm diam. leading to foliage distortions and, when abundant to leaf drop. *Spermogonial morph lesions* as for sexual morph. *Asexual morph lesions*, leaf spots of variable shape, subcircular to elongating along leaf veins to angular or irregular 1–7 × 1–2.5 mm, greyish brown to black, scattered over lamina, sometimes somewhat raised, coalescing with age and leading to premature leaf drop. *Internal mycelium*, 3–6 µm diam, branched, septate, hyaline to pale brown, smooth.

*Ascomata* pseudothecial, superficial, epiphyllous, in large erumpent ascostromata having a foot-like hypostroma, spherical, 128–165 × 90–192 µm, walls of brown *textura angularis*, 6–9 cells, 42–57.5 µm thick, smooth. *Dehiscence* ostiolate, 2–10 µm in diam; *Asci* bitunicate, clavate, 66.5–90 × 13–16.5 µm, 8-spored. *Ascospores* ellipsoidal, 15–20 × 4–5 µm, 1-septate, constricted at septum, hyaline, smooth. *Conidiophores* amphigenous, sometimes emerging from a thin layer of brown cells or ill-developed stroma, mostly reduced to conidiogenous cells, sparse or subfasciculate to forming dense parallel groups, cylindrical, bulbose at the base, erect, straight or slightly flexuous to geniculate torward the apices, unbranched, 31–56 × 4–6 µm, 0–1 septate, pale brown, smooth. *Conidiogenous cells* holoblastic, integrated, cylindrical to subcylindrical, terminal, proliferating sympodially with 1–3 loci, 2 µm diam, flat, unthickened, not darkened. *Conidia* solitary, obclavate, straight to usually curved or twisted into a somewhat sigmoid shape, 27.5–62 × 6–11 µm, apex rounded, base attenuated to a truncate hilum, 0–1-septate, somewhat constricted at the septum, subhyaline to pale brown, smooth to somewhat roughened, thin-walled, hilum 2 µm wide, unthickened, not darkened. *Spermogonia* adaxial superficial, in stromatic groups, globose, 112.5–138 × 87.5–150 µm, walls of pale to dark brown *textura angularis*, 4–8 cells-thick, 37.5–92.5 µm, ostiolate, smooth. *Spermatiophores* phialidic, lageniform, integrated, 10–16 × 1–2 µm, hyaline, smooth. *Spermatia* dumb-bell-shaped, 7–4 × 1 µm, aseptate, hyaline, smooth.

**Hosts and Distribution**: on *Hevea* spp. (Euphorbiaceae), *H. benthamiana* (Brazil); *H. brasiliensis* (Bolivia, Brazil, Colombia, Costa Rica, French Guyana, Guatemala, Guyana, Honduras, México, Nicaragua, Panamá, Peru, South America, Suriname, Trinidad & Tobago, Venezuela); *H.colina* (Brazil); *H. confusa* (Guyana); *H. guyanensis* (Brazil, Guyana, South America, Suriname); *H. guianensis* (South America); *H. guianensis var. lutea* (Peru); *H. lutea* (Peru); *H. paludosa* (Brazil); *H. randiana* (Brazil); *H. spruceana* (Brazil, Costa Rica, Guyana, Panama, South America, Suriname, Trinidad & Tobago); *Hevea* spp. (Brazil, Trinidad & Tobago) [47], [48].

***Material examined*** **: sexual morph** and **spermogonial morph** - BRAZIL, Pará, Belém, on living leaves of *Hevea brasiliensis*, 14 March 2007, H. C. Evans (VIC 30547), **asexual morph** - Bahia, Porto Seguro, on living leaves of *Hevea brasiliensis*, September 2008, B. T. Hora Junior (VIC 39722 – COAD 1339)

***Additional material examined***: **sexual morph** - BRAZIL, Pará, Belém, 14 March 2007, H. C. Evans (VIC 30549), Acre, Xapuri, January 2010, B. T. Hora Junior (VIC 39728); Minas Gerais, Oratórios, Fazenda Experimental EPAMIG, September 2010, B. T. Hora Junior (VIC 39729); **asexual morph** - BRAZIL, Rondônia, Buritis, July 2010, J. Honorato Junior (VIC 39723 – COAD 1340); Mato Grosso, Itiquira, February 2009, B. T. Hora Junior (VIC 39724 – COAD 1341); Espírito Santo, Sooretama, December 2010, B. T. Hora Junior (VIC 39725 – COAD 1342); **spermogonial morph** - BRAZIL, Rondônia, Ariquemes, on living leaves of *Hevea brasiliensis*, July 2010, J. Honorato Junior (VIC 39726); Espírito Santo, Cachoeiro do Itapemirim, December 2010, B. T. Hora Junior (VIC 39727).

## Discussion

DNA sequences of the three morphs in the life cycle of *M. ulei* collected over a wide geographic area in Brazil confirmed the asexual:sexual morph connection of this fungal species, but the current classification of the pathogen in the Planistromellaceae [13], [14] was not supported by any of the phylogenies. The analysis conducted in the present study was based on nuclear and mitochondrial ribosomal rDNA, as well as on protein-coding genes, and supports the classification of *M. ulei* in the family Mycosphaerellaceae s. str., in the order Capnodiales, class Dothideomycetes of the phylum Ascomycota. Mycosphaerellaceae is a well-supported family within the Capnodiales with *Mycosphaerella punctiformis* (*Ramularia endophylla* as anamorph) as the type species [49], [50]. DNA sequence data of *R. endophylla* were included in all analyses of generic relationships, corroborating the classification of the pathogen at the family level and revealing a close relationship of the SALB fungus with *Mycosphaerella*. Currently, robust multi-gene phylogenetic analysis concluded that *Mycosphaerella* is a polyphyletic group [51]–[53], suggesting that *Mycosphaerella* s. lat. should be subdivided to reflect natural groups (genera) as defined by their asexual morphs, since *Mycosphaerella* s. str. is restricted to species with *Ramularia* morphs [52].

The phylogeny of species representing the core genera of the Planistromellaceae formed a clade within the order Botryosphaeriales and revealed that previous morphology-based definitions of genera have resulted in an artificial classification system and thus, the genera *Planistromella* and *Planistroma* have been considered to constitute one genus, namely *Kellermania* [15]. In addition to *M. ulei*, other species previously classified in Planistromellaceae have been transferred to the Mycosphaerellaceae, as in the case of *Eruptio acicula* [54] and to the Phaeosphaeriaceae as for *Loratospora aestuarii* [53], after phylogenetic re-evaluation. The classification of *Microcyclus* as a mycosphaerella-like organism has been discussed previously [55]. The *Microcyclus* genus has ellipsoidal, hyaline, 1-septate ascospores in clavate, bitunicate asci, typical of the genus *Mycosphaerella* Johanson [26]. The development of stromatic tissue in *Microcyclus* appears to be the only character that contributes to its separation from the genus *Mycosphaerella* [55]; although Barr [13] further characterized the genus by the presence of periphysate ostioles in the type species *Microcyclus angolensis*. Nevertheless, some mycosphaerella-like species have similar strongly erumpent ascostromata [12], [26]. As already demonstrated for *Mycosphaerella*, the genus *Microcyclus*, as currently circumscribed, also appears to be polyphyletic, given the variety of asexual morphs associated with the assigned species [55].

The conidial morph of *M. ulei, F. heveae*, was “tentatively retained in *Fusicladium* since it is morphologically indistinguishable from other species of this genus” [25]. Bonorden [56] characterized the genus *Fusicladium* as having denticulate conodiogenous cells. In Saccardo [57], Lindau [58] and Ferraris [59] this genus was described as having sympodial conidiogenous cells (denticulate) or percurrent (annelidic). In the 1950s, Hugues [60] limited *Fusicladium* spp. to species having conidiogenous cells with sympodial proliferation. Sympodial proliferation on its own is a character that is far from adequate for grouping species within the cercosporoid complex as this is a feature widespread among genera of cercosporoids. Furthermore, our observations have consistently shown that *P. ulei* does not have denticulate conidiogenous loci but instead it has the typical locus structure of fungi in *Pseudocercospora* - truncate without thickening and pigmentation of conidiogenous scars contrarily to “slightly denticulate” as in the description included in Schubert et al. [25]. Another feature consistently observed that was in disagreement with Schubert et al. [25] as the absence of “well formed stromata from which the conidiophores emerge”. Instead, only a 1–2 cell layer of pseudoparenchymatous cells from which a dense palisade of somewhat parallel to subfasciculate conidiophores was observed. Species of *Fusicladium* s. lat. form a monophyletic group in the Venturiaceae [24], [61], whilst other fusicladium-like species have been assigned to the Sympoventuriaceae, both in the Venturiales [24]. Although *F. heveae* has already been treated as a species of the *Passalora*-type and, therefore, a cercosporoid fungus [16], our molecular data demonstrate that the SALB pathogen is better accommodated in the clade *Pseudocercospora* s. str., as defined by Crous et al. [52], [62] in which the type species, *Pseudocercospora vitis*, resides (Figures 1 and 4). Whilst in the Mycosphaerellaceae, many asexual morphs evolved in more than one clade and thus represent different genera [51], [52], the morphological convergence of ‘*F. heveae*’ is at the order level, as is also evident for ‘*A. ulei*’ (Figure 1).

*Pseudocercospora* s. str. is a well-defined genus in the Mycosphaerellaceae, based on both DNA sequence and morphological data [52], [62], which is now utilized as a holomorph name with species having mycosphaerella-like sexual morphs. As observed for ‘*F. heveae*’ in the present study, when the phylogenetic species concept is applied to other species of the genera *Cercostigmina*, *Phaeoisariopsis* and *Stigmina*, they are reduced to synonymy with the genus *Pseudocercospora* [62]–[65].

*Pseudocercospora* s. str. includes several well-known and highly destructive plant pathogens affecting important crops worldwide [62]. Recognizing the SALB fungus as belonging to such genus allows for the adoption of comparative epidemiology and genomics approaches, using better studied pathogenic species such as *P. fijiensis*, the causal agent of the black leaf streak (black Sigatoka) disease of banana [66].

Based on the molecular evidence connecting the three spore morphs in the life cycle of the SALB fungus and comparative biology from phylogenetic relationships, the life cycle of *M. ulei* was then re-assessed with special attention to its intermediate ‘pycnidial’ morph. Physiological data indicate that leaves at the B and C stages act as sinks with high respiration rates and are almost lignin-free [3]. Previous studies report that conidial lesions are the first stage of the disease and fertile pycnidia occur three to five weeks later on mature or near-mature diseased leaves [2], [19]. Ascostromata become mature at about four to six weeks and the formation of ascospores is correlated with effete pycnidia [1]. In our study, mature ‘pycnidia’ were seen after two to three weeks on the upper surface of leaves in the C/D and D stages in the area previously occupied by abaxial conidial (*Pseudocercospora*) lesions. After four weeks, the ascostromata became more visible and increased in number and size.

In contrast to a previous study [1], but in accordance with an earlier study [19], our results confirmed that the pycniospores do not germinate *in vitro* and fail to infect rubber leaves. These observations corroborate the hypothesis that the supposedly erumpent pycnidial structures are in fact spermogonia and are likely to be involved in the initial stages of the sexual cycle, as suggested previously [2], [19], [26]. Commonly, fungi in the Mycosphaerellaceae produce spermogonia and the spermatia are thought to act as male sexual elements because of their small size and inability to germinate and to infect the host plant. Pseudothecial development begins from protoascomata, usually concurrently with the spermogonia, and the two structures are similar in size and shape [67]–[70]. The production of spermatia is also part of the life cycle of *Pseudocercospora fijiensis* [66], [71], and such spermatia are considered as male gametes, formed in spermogonia, which usually develop from the substomatal chambers before the formation of pseudothecia; although the cytological details of spermatization and ascospore development have not yet been elucidated [66]. Similar fertilization events can also take place in *P. ulei*.

**Figure 6:**
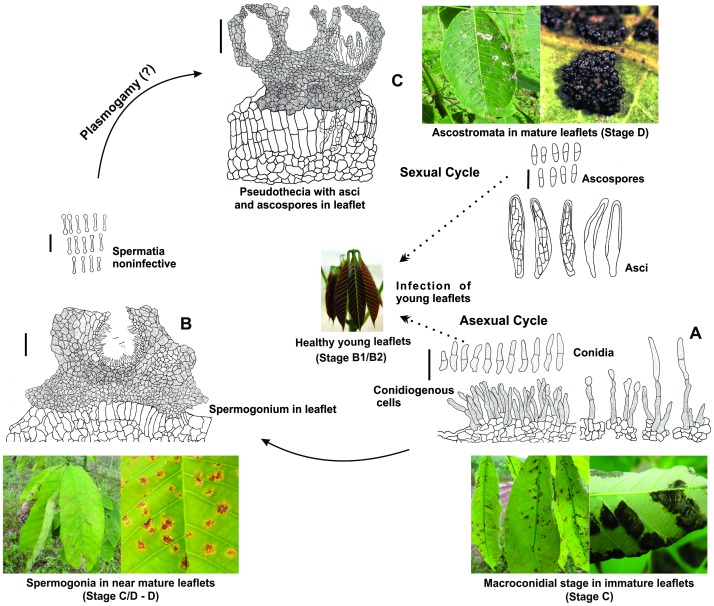
Hypothetical life cycle of *Pseudocercospora ulei*. **A**. Asexual morph with conidiophores and conidia (Bar =  35µm) and conidiogenous cells with conidia at different stages of conidial formation. Pictures: Lesions to which the asexual morph is associated (left) and close-up of leaf bearing typical lesions (right). **B**. Spermogonial morph with stroma, spermogonia (Bar =  30 µm) and spermatia (Bar =  7 µm). Pictures: Lesions to which the spermogonial morph is associated (left), and close-up of the same lesions (right). **C**. Sexual morph with stroma, pseudothecia, asci and ascospores (Bar = 60µm). Pictures: Lesions to which the sexual morph is associated (left), and close-up of stromata (right). Dotted arrows indicate that both ascospores and conidia can infect young leaves.

A revised version of the life cycle of this pleomorphic fungus is presented (Figure 6). Only one asexual morph, which belongs to *Pseudocercospora* s. str., is present and conidia infect young leaves being responsible for the destructive secondary disease cycles in the field. The sexual cycle begins with spermogonial developing in the leaf (from stage C/D) and finishes with mature ascospores in pseudothecia within pronounced, erumpent ascostromata of the *Mycosphaerella*-type.

The persistence of significant gaps in the knowledge on the biology of a fungus of the importance of *P. ulei* has puzzled Money [7] who stated “I am astonished by the apparent ostrich-like behavior of the rubber-manufacturing companies towards the disease… the scientists endeavor to understand the fungus appears frozen… the lack of recent publications in the public domain is remarkable.” His anxiety was clearly justifiable considering the lack of a proper taxonomic treatment for the fungus and of adequate understanding of its life cycle as indicated by the present findings. Much more needs to be investigated about the SALB fungus if we are willing to properly understand the biology of the fungus and prepare to deflect the threats represented by SALB.

## Supporting Information

Table S1 **GenBank accession numbers of sequences derived from strains used in the phylogenetic analysis.** Newly deposited sequences are shown in bold.
(DOCX)

## References

[1] Holliday P (1970) South American leaf blight (*Microcyclus ulei*) of *Hevea brasiliensis* . Commonwealth Mycological Institute Phytopath Pap 12: 1–31.

[2] Chee KH, Holliday P (1986) South American leaf blight of *Hevea* rubber. Malaysian Rubber Research and Development Board. Malaysian Rubber Research and Development Board Monograph No. 13. 50 p.

[3] Lieberei R (2007) South American Leaf Blight of the rubber tree (*Hevea* spp.): New steps in plant domestication using physiological features and molecular markers. Ann Bot 100: 1125–1142. 17650512 10.1093/aob/mcm133 PMC2759241

[4] van Beilen JB, Poirier Y (2007) Establishment of new crops for the production of natural rubber. Trends Biotechnol 25: 522–529. 17936926 10.1016/j.tibtech.2007.08.009

[5] Davis W (1997) One river: Science, adventure and hallucinogenics in the Amazon Basin. London: Simon and Schuster. 537p.

[6] Grandin G (2009) Fordlandia: the rise and fall of Henry Ford's forgotten jungle city. New York: Metropolitan Books. 183p.

[7] Money NP (2007) The triumph of the fungi: A Rotten History. New York: Oxford University Press. 197p.

[8] Gasparotto L, Santos AF, Pereira JCR, Ferreira FA (1997) Doenças da seringueira no Brasil. Embrapa-SPI: Manaus: Embrapa-CPAA. 168p.

[9] Hennings P (1904) Uber die auf *Hevea* –arten bisher beobachteten parasitischen pilze. Notizbl bot Gart Mus Berl 4: 133–139.

[10] Müller E, Arx JA von (1962) Die Gattungen der didymosporen Pyrenomyceten. *Beitr. Kryptog* . Flora Schweiz 11: 1–992.

[11] Arx JA von (1983) *Mycosphaerella* and its anamorphs. Proc Konink Nederl Acad Wetensch 86: 15–54.

[12] Evans HC (1984) The genus *Mycosphaerella* and its anamorphs *Cercoseptoria*, *Dothistroma* and *Lecanosticta* on pines. Mycol Pap 153: 1–102.

[13] Barr ME (1996) Planistromellaceae, a new family in the Dothideales. Mycotaxon 60: 433–442.

[14] Lumbsch HT, Huhndorf SM (2010) Outline of Ascomycota– 2009. Fieldiana Life Earth Sci 1: 1–64.

[15] Minnis AM, Kennedy AH, Grenier DB, Palm ME, Rossman AY (2012) Phylogeny and taxonomic revision of the Planistromellaceae including its coelomycetous anamorphs: contributions towards a monograph of the genus *Kellermania* . Persoonia 29: 11–28 10.3767/003158512X658766 23606762 PMC3589788

[16] Crous PW, Braun U (2003). *Mycosphaerella* and its anamorphs. 1. Names published in *Cercospora* and *Passalora*. CBS Biodiversity Series 1: 1–571. Centraalbureau voor Schimmelcultures, Utrecht, Netherlands.

[17] Mugambi GK, Huhndorf SM (2009) Molecular phylogenetics of Pleosporales: Melanommataceae and Lophiostomataceae re-circumscribed (Pleosporomycetidae, Dothideomycetes, Ascomycota). Stud Mycol 64: 103–121 10.3114/sim.2009.64.05 20169025 10.3114/sim.2009.64.05 PMC2816968

[18] Zhang Y, Crous PW, Schoch CL, Hyde KD (2012) Pleosporales. Fungal Divers. 53: 1–221. doi 10.1007/s13225-011-0117-x 10.1007/s13225-011-0117-x PMC3477819 23097638

[19] Langford MH (1945) South American leaf blight of *Hevea* rubber trees. Technical Bulletin United States Department of Agriculture.882: 31p

[20] Guyot J, Doaré F (2010) Obtaining isolates of *Microcyclus ulei*, a fungus pathogenic to rubber trees, from ascospores. J Plant Pathol 92: 765–768.

[21] Guyot J, Condina V, Doaré F, Cilas C, Sache I (2013). Role of ascospores and conidia in the initiation and spread of South American Leaf Blight in a rubber tree plantation. Plant Pathol. doi: 10.1111/ppa.12126.

[22] Arx JA von, Müller E (1975) A re-evaluation of the bitunicate ascomycetes with keys to families and genera. Stud Mycol 9: 1–159.

[23] Eriksson OE, Hawksworth DL (1993) Outline of the ascomycetes-1993. Syst Ascomycetum 12: 51–257.

[24] Zhang Y, Crous PW, Schoch CL, Bahkali AH, Guo LD, et al (2011) A molecular, morphological and ecological re-appraisal of Venturiales - a new order of Dothideomycetes. Fungal Divers 51: 249–277. 22368534 10.1007/s13225-011-0141-x PMC3285419

[25] Schubert K, Ritschel A, Braun U (2003) A monograph of *Fusicladium* s. lat. (Hyphomycetes). Schlechtendalia 9: 1–132.

[26] Evans HC (2002) Invasive neotropical pathogens of tree crops. Pages 83–112 in: Tropical Mycology: Vol. 2, Micromycetes. R. Watling, J. Frankland, M. Ainsworth, S. Isaac, and C. Robinson, eds. Oxon, UKCABI Publishing, Wallingford

[27] Wingfield MJ, Beer de ZW, Slippers B, Wingfield BD, Groenewald JZ, et al (2011) One fungus, one name promotes progressive plant pathology. Mol Plant Pathol 13: 604–613 10.1111/j.1364-3703.2011.00768.x 22146077 PMC6638803

[28] Kirk PM, Cannon PF, Minter DW, Stalpers JA (2008) Dictionary of the Fungi. 10th ed. Wallingford: CABI. ISBN 0-85199-826-7.

[29] Junqueira NTV, Chaves GM, Zambolim L, Romeiro RS, Gasparotto L (1984) Isolamento, cultivo e esporulação de *Microcyclus ulei*, agente etiológico do mal das folhas da seringueira. Rev Ceres 31: 322–331.

[30] Doyle JJ, Doyle JL (1990) Isolation of plant DNA from fresh tissue. Focus 12: 13–15.

[31] Rehner SA, Samuels GJ (1994) Taxonomy and phylogeny of *Gliocladium* analysed from nuclear large subunit ribosomal DNA sequences. Mycol Res 98: 625–634.

[32] Vilgalys R, Hester M (1990) Rapid genetic identification and mapping of enzymatically amplified ribosomal DNA from several *Cryptococcus* species. J Bacteriol 172: 4238–4246. 2376561 10.1128/jb.172.8.4238-4246.1990 PMC213247

[33] White TJ, Bruns T, Lee S, Taylor J (1990) Amplification and direct sequencing of fungal ribosomal RNA genes for phylogenetics. InInnis MGelfand DHSninsky JJWhite TJSan DiegoPCR protocols. Academic Press315322

[34] Li KN, Rouse DI, German TL (1994) PCR primers that allow intergenic differentiation of ascomycetes and their application to *Verticillium* spp. Appl Environ Microbiol 60: 4324–4331. 7811072 10.1128/aem.60.12.4324-4331.1994 PMC201988

[35] Schmitt I, Crespo A, Divakar PK, Frankhauser JD, Herman-Sackett E, et al (2009) New primers for promising single-copy genes in fungal phylogenetics and systematics. Persoonia 23: 35–40 10.3767/003158509X470602 20198159 PMC2802727

[36] Carbone I, Kohn LM (1999) A method for designing primer sets for speciation studies in filamentous ascomycetes. Mycologia 91: 553–555.

[37] Staden R (1996) The staden sequence analysis package. Mol. Biotechnol 5: 233–241. 8837029 10.1007/BF02900361

[38] Grigoriev IV, Nordberg H, Shabalov I, Aerts A, Cantor M, et al (2012) The genome portal of the Department of Energy Joint Genome Institute. Nucleic Acids Res 40: 26–32 10.1093/nar/gkr947 PMC3245080 22110030

[39] Edgar RC (2004) MUSCLE: multiple sequence alignment with high accuracy and high throughput. Nucleic Acids Res 32: 1792–1797 10.1093/nar/gkh340 15034147 PMC390337

[40] Tamura K, Peterson D, Peterson N, Stecher G, Nei M, et al (2011) MEGA5: molecular evolutionary genetics analysis using maximum likelihood, evolutionary distance, and maximum parsimony methods. Mol Biol Evol 28: 2731–2739 10.1093/molbev/msr121 21546353 PMC3203626

[41] Ronquist F, Huelsenbeck JP (2003) MrBayes 3: Bayesian phylogenetic inference under mixed models. Bioinformatics 19: 1572–1574. 12912839 10.1093/bioinformatics/btg180

[42] Nylander JAA (2004) MrModeltest v 2.2. Program distributed by the author. Uppsala University, Uppsala, SwedenEvolutionary Biology Centre

[43] Rambaut A, Drummond AJ (2007) Tracer v1.4. Available: http://beast.bio.ed.ac.uk/Tracer

[44] Crous PW, Gams W, Stalpers JA, Robert V, Stegehuis G (2004) MycoBank: an online initiative to launch mycology into the 21st century. Stud Mycol 50: 19–22.

[45] Halleé F, Martin R (1968) Eétude de la croissance rythmique chez l′heéveéa (*Hevea brasiliensis* Mull. Arg., Euphorbiaceées, Crotonoiédeées). Adansonia 8: 475–503.

[46] Junqueira NTV, Chaves GM, Zambolim L, Alfenas AC, Gasparotto L (1988) Reacãao de clones de seringueira a vaários isolados de *Microcyclus ulei* . Pesq Agropec Bras 23: 877–893.

[47] Holliday P (1970) *Microcyclus ulei* . CMI Descriptions of pathogen fungal and bactéria 410: 1–2.

[48] Farr DF, Rossman AY (2013) Fungal Databases, Systematic Mycology and Microbiology Laboratory, ARS, USDA. Available: http://nt.ars-grin.gov/fungaldatabases/. Accessed 19 Sep 2013.

[49] Verkley GJM, Crous PW, Groenewald JZ, Braun U, Aptroot A (2004) *Mycosphaerella punctiformis* revisited: morphology, phylogeny, and epitypification of the type species of the genus *Mycosphaerella* (Dothideales, Ascomycota). Mycol Res 108: 1271–1282 10.1017/S0953756204001054 15587478

[50] Crous PW, Schoch CL, Hyde KD, Wood AR, Gueidan C, et al (2009) Phylogenetic lineages in the Capnodiales. Stud Mycol 64: 17–47 10.3114/sim.2009.64.02 20169022 PMC2816965

[51] Crous PW, Braun U, Groenewald JZ (2007) *Mycosphaerella* is polyphyletic. Stud Mycol 58: 1–32 10.3114/sim.2007.58.01 18490994 PMC2104738

[52] Crous PW, Summerell BA, Carnegie AJ, Wingfield MJ, Hunter GC, et al (2009) Unravelling *Mycosphaerella*: do you believe in genera? Persoonia 23: 99–118 10.3767/003158509X479487 20198164 PMC2802725

[53] Schoch CL, Sung GH, Lopez-Giraldez F, Townsend JP, Miadlikowska J, et al (2009) The Ascomycota tree of life: A phylum-wide phylogeny clarifies the origin and evolution of fundamental reproductive and ecological traits. Syst Biol 58: 224–239 10.1093/sysbio/syp020 20525580

[54] Verkley GJM, Starink-Willemse M, Iperen A van, Abeln ECA (2004) Phylogenetic analyses of *Septoria* species based on the ITS and LSU-D2 regions of nuclear ribosomal DNA. Mycologia 96: 558–571. 21148878

[55] Cannon PI, Camaran CC, Romero AI (1995) Studies on biotrophic fungi from Argentina: *Microcyclus porleriae*, with a key to South American species of *Microcyclus* . Mycol Res 99: 353–356.

[56] Bonorden HF (1851) Handbuch der allgemeinen Mykologie. Stuttgart.

[57] Saccardo PA (1897) Sylloge Fungorum vol. 12 (Sydow, P. ed.). Borntraeger, Berlin.

[58] Lindau G (1907) Dr. L. Rabenhorst's Kryptogamen-Flora von Deutschland, Oesterreich und der Schweiz. Zweite Auflage. Erster Band: Pilze. Die Pilze Deutschlands, Oesterreichs und der Schweiz. VIII. Abteilung: Fungi imperfecti: Hyphomycetes (erste Halfte), Mucedinaceae, Dematiaceae (Phaeosporae und Phaeodidymae). Leipzig.

[59] Ferraris T (1912) Hyphales, Tuberculariaceae - Stilbaceae. Flora Italica Cryptogama Pars I: Fungi, Fasc 6: 195–534.

[60] Hughes SJ (1953) Some foliicolous hyphomycetes. Can J Bot 31: 565–576.

[61] Beck A, Ritschel A, Schubert K, Braun U, Triebel D (2005) Phylogenetic relationships of the anamorphic genus *Fusicladium* s. lat. as inferred by ITS nrDNA data. Mycol Prog 4: 111–116 10.1007/s11557-006-0114-8

[62] Crous PW, Braun U, Hunter GC, Wingfield MJ, Verkley GJM, et al (2013) Phylogenetic lineages in *Pseudocercospora* . Stud Mycol 75: 37–114 10.3114/sim0005 24014898 PMC3713886

[63] Crous PW, Kang JC, Braun U (2001) A phylogenetic redefinition of anamorph genera in *Mycosphaerella* based on ITS rDNA sequence and morphology. Mycologia 93: 1081–1101 10.3114/sim.55.1.163

[64] Braun U, Hill CF (2002) Some new micromycetes from New Zealand. Mycol Prog 1: 19–30 10.1007/s11557-006-0002-2

[65] Crous PW, Liebenberg MM, Braun U, Groenewald JZ (2006) Re-evaluating the taxonomic status of *Phaeoisariopsis griseola*, the causal agent of angular leaf spot of bean. Stud Mycol 55: 163–173 10.3114/sim.55.1.163 18490977 PMC2104728

[66] Churchill ACL (2011) *Mycosphaerella fijiensis*, the black leaf streak pathogen of banana: progress towards understanding pathogen biology and detection, disease development, and the challenges of control. Mol Plant Pathol 12: 307–328 10.1111/j.1364-3703.2010.00672.x 21453427 PMC6640443

[67] Higgins BB (1920) Morphology and life history of some Ascomycetes with special reference to the presence and function of spermatia. Am J Bot 7: 435–445.

[68] Snyder WC (1946) Spermogonia versus pycnidia in *Mycosphaerella brassicicola* . Phytopathology 36: 481–484.

[69] Dring D (1961) Studies on *Mycosphaerella brassicicola* (Duby) Oudem. T Brit Mycol Soc 44: 253–264.

[70] Inman AJ, Sivanesan A, Fitt BDL, Evans RL (1991) The biology of *Mycosphaerella capsellae* sp. nov., the teleomorph of *Pseudocercosporella capsellae*, cause of white leaf spot of oilseed rape. Mycol Res 95: 1334–1342.

[71] Liberato JR, Peterson RA, Gasparotto L, Ferrari JT, Grice K, et al. (2009) Black sigatoka of banana (*Mycosphaerella fijiensis*). Pest and Diseases Image Library, Species Content Page. Available: http://www.padil.gov.au/viewPestDiagnosticImages.aspx?id=431. Plant Biosecurity Toolbox/Info Sheet. Available: http://www.padil. gov.au/pbt/index.php?q = node/46&pbtID = 166.

