# Supplementary material for: Erasing the Past: A New Identity for the Damoclean Pathogen Causing South American Leaf Blight of Rubber
Source: PLoS One. 2014 Aug 15;9(8):e104750. doi: 10.1371/journal.pone.0104750 (PMC4134235; doi:10.1371/journal.pone.0104750)
Supplement: Table S1 — GenBank accession numbers of sequences derived from strains used in the phylogenetic analysis. Newly deposited sequences are shown in bold. (DOCX) [file pone.0104750.s001.docx]

**Table S1.** GenBank accession numbers of sequences derived from strains used in the phylogenetic analysis. Newly deposited sequences are shown in bold.

| **Species** | **S****train^1^** | **LSU** | **mtSSU** | **MCM7** | **ITS** | **Act** | **Tef1** |
| --- | --- | --- | --- | --- | --- | --- | --- |
| *Alternaria alternata* | CBS 916.96, H02-747S-5 | — | EF152432 | JN672967 | — | — | — |
| *Alternaria brassicicola* | ATCC 96836 | — | — | Genome^2^ | — | — | — |
| *Apiosporina collinsii* | CPC 12229, CBS 11893 | EU035443 | — | JN672970 | — | — | — |
| *Aposphaeria corallinolutea* | PD 83/367, PD 83/831 | JF740329 JF740330 | — | — | — | — | — |
| *Aposphaeria populina* | CBS 543.70, CBS 350.82 | EU754130 EU035443 | — | — | — | — | — |
| *Aspergillus niger* | CBS 513.88, ATHUM 5044 | AM270051 | EU982148 | Genome | — | — | — |
| *Capnodium coffeae* | CBS 147.52, AFTOL-ID 939 | GU214400 | FJ190609 | — | — | — | — |
| *Cercospora apii* | CBS 118712 | GQ852583 | — | — | — | — | — |
| *Cercospora beticola* | 21 | — | DQ028504 | — | — | — | — |
| *Cercospora capsici* | CBS 118712 | GU214653 | — | — | — | — | — |
| *Cercospora coffeicola* | 172 | — | DQ028515 | — | — | — | — |
| *Cercospora* sp. | CBS 113997, CPC 10550 | — | JX142504 | JX142475 | — | — | — |
| *Cercospora zebrina* | CBS 118790 | GU214657 | — | — | — | — | — |
| *Cercospora zeae-maydis* | SCOH1-5 | — | — | Genome | — | — | — |
| *Cladosporium cladosporioides* | CBS 109.21, AFTOL-ID 1289 | EU019262 | FJ190628 | — | — | — | — |
| *Cladosporium oxysporum* | CBS 125.80 | — | DQ089646 | — | — | — | — |
| *Cladosporium sphaerospermum* | UPSC 957 | — | DQ089641 | — | — | — | — |
| *Cladosporium uredinicola* | CPC 5390 | EU019264 | — | — | — | — | — |
| *Cladosporium variabile* | CGMCC 3.4011 | — | DQ089643 | — | — | — | — |
| *Cochliobolus heterostrophus* | AFTOL-ID 54, C4 | — | AY544737 | Genome | — | — | — |
| *Conidioxyphium gardeniorum* | CPC 14327 | GU301807 | — | — | — | — | — |
| *Davidiella allicina* | CBS 723.79 | **KC800751** | **KC800778** | — | — | — | — |
| *Davidiella tassiana* | CBS 723.79, CPC 11600 | GU214410 | EU514455 | — | — | — | — |
| **Table S1.** Continued |  |  |  |  |  |  |  |
| **Species** | **Strain** | **LSU** | **mtSSU** | **MCM7** | **ITS** | **Act** | **Tef1** |
| *Delphinella strobiligena* | AFTOL-ID 1257 | DQ470977 | — | — | — | — | — |
| *Dissoconium aciculare* | CBS 204.89 | GU214419 | — | — | — | — | — |
| *Dissoconium dekkeri* | CPC 1232 | GU214423 | — | — | — | — | — |
| *Dissoconium musae* | CBS 122453, CBS 122454 | — | EU514402 EU514403 | — | — | — | — |
| *Dothidea berberitis* | CBS 187.58 | **KC800752** | **KC800780** | — | — | — | — |
| *Dothidea insculpta* | CBS 189.58, AFTOL-ID 921 | DQ247802 | FJ190602 | — | — | — | — |
| *Dothidea sambuci* | AFTOL-ID 274 | DQ470984 | AY544739 | — | — | — | — |
| *Dothiora cannabinae* | AFTOL-ID 1359 | DQ470984 | FJ190636 | — | — | — | — |
| *Dothistroma pini* | CBS 116487 | GU214426 | — | — | — | — | — |
| *Dothistroma septosporum* | NZE10 | — | — | Genome | — | — | — |
| *Fusicladium catenosporum* | CBS 447.91 | EU035427 | — | — | — | — | — |
| *Fusicladium mandshuricum* | CBS 112235 | EU035433 | — | — | — | — | — |
| *Fusicladium oleagineum* | CBS 113427 | EU035434 | — | — | — | — | — |
| *Fusicladium phillyreae* | CBS 113539 | EU035435 | — | — | — | — | — |
| *Fusicladium pomi* | UFVVi-235, UFVVi-373 | **KC800753 KC800754** | **KC800781** **KC800782** AF051644 | **KC800766 KC800767** | — | — | — |
| *Lecanosticta pini* | CBS 871.95 | GQ852598 | — | — | — | — | — |
| *Leptosphaeria dryadis* | CBS 473.64 | — | — | — | — | — | — |
| *Leptosphaeria macrospora* | Kruys 501 (UPS) | — | DQ384089 | — | — | — | — |
| *Leptoxyphium fumago* | CBS 123.26 | GU301831 | — | — | — | — | — |
| *Lophiostoma arundinis* | AFTOL-ID 1606, KT 651 | DQ782384 | — | JN993405 | — | — | — |
| *Lophiostoma compressum* | KT 521 | — | — | JN993399 | — | — | — |
| *Lophiostoma macrostomum* | Lundqvist 20504, KT 709 | — | DQ384088 | JN993403 | — | — | — |
| **Table S1.** Continued |  |  |  |  |  |  |  |
| ***Species*** | **Strain** | **LSU** | **mtSSU** | **MCM7** | **ITS** | **Act** | **Tef1** |
| *Lophiostoma semiliberum* | KT 828 | — | — | JN993400 | — | — | — |
| *Lophiostoma winteri* | KT 764 | — | — | JN993401 | — | — | — |
| *Metacoleroa dickiei* | Kruys 503 (UPS) | DQ384100 | DQ384080 | — | — | — | — |
| *Microxyphium citri* | CBS 451 66 | GU301848 | AF346421 | — | — | — | — |
| *Pallidocercospora acaciigena* | CPC 3838 | GU214661 | — | — | — | — | — |
| *Pallidocercospora heimii* | CBS 110682 | GQ852604 | — | — | — | — | — |
| *Pallidocercospora heimioides* | CBS 111364 | DQ204752 | — | — | — | — | — |
| *Pallidocercospora irregulariramosa* | CBS 111211 | GQ852609 | — | — | — | — | — |
| *Pallidocercospora konae* | CBS 120748 | GU253852 | — | — | — | — | — |
| *Passalora eucalypti* | CBS 111318, CPC 1457 | GQ852620  GU214458 | — | — | GU269845 | GU320548 | GU384558 |
| *Phaeophleospora eugeniicola* | CPC 2558 | FJ493209 | — | — | — | — | — |
| *Pleospora herbarum* | AFTOL-ID 940 | — | FJ190610 | — | — | — | — |
| *"Pseudocercospora" colombiensis* | CMW 11255 | DQ204745 | — | — | — | — | — |
| *"Pseudocercospora" thailandica* | CBS 116367, X22 | GU253837 | EU514439 | — | — | — | — |
| *Pseudocercospora acericola* | CBS 122279 | — | — | — | GU269650 | GU320358 | GU384368 |
| *Pseudocercospora angolensis* | CBS 112933, CPC 4118 | GU214470 | — | — | GU269836 | JQ325010 | GU384548 |
| *Pseudocercospora assamensis* | CBS 122467 | EU514445 | — | — | — | — | — |
| *Pseudocercospora basitruncata* | CBS 114664 | — | — | — | DQ267600 | DQ147622 | DQ211675 |
| *Pseudocercospora chengtuensis* | CPC 10696 | — | — | — | GU269673 | GU320379 | GU384390 |
| **Table S1.** Continued |  |  |  |  |  |  |  |
| **Species** | **Strain** | **LSU** | **mtSSU** | **MCM7** | **ITS** | **Act** | **Tef1** |
| *Pseudocercospora cladosporioides* | CBS 117482 | — | — | — | GU269678 | GU320383 | GU384395 |
| *Pseudocercospora eucalyptorum* | CBS 116359 | — | — | — | GU269812 | GU320514 | GU384524 |
| *Pseudocercospora eumusae* | CBS 121383 | — | EU514416 | — | — | — | — |
| *Pseudocercospora fijiensis* | CBS 120258, CIRAD 86, UFVMf-119 | DQ678098 KC800749 | FJ190656 KC800776 | Genome KC800763 | EU514248 | Genome | Genome |
| *Pseudocercospora fuligena* | CPC 12296 | — | — | — | GU269711 | GU320415 | GU384427 |
| *Pseudocercospora gracilis* | CBS 243.94 | — | — | — | DQ267582 | DQ147616 | DQ211666 |
| *Pseudocercospora griseola f. griseola* | CPC 10461, CBS 119112 | GU348997 | — | — | GU269717 | GU320421 | GU384433 |
| *Pseudocercospora humulicola* | CPC 10049 | — | — | — | GU269724 | JQ325018 | JQ324996 |
| *Pseudocercospora indonesiana* | CBS 122473 | — | EU514446 | — | — | — | — |
| *Pseudocercospora kaki* | MUCC 900 | — | — | — | GU269729 | GU320431 | GU384442 |
| *Pseudocercospora longispora* | CBS 122470, CBS 122470 | — | EU514448 | — | GU269734 | GU320436 | GU384447 |
| *Pseudocercospora luzardii* | CPC 2556 | — | — | — | GU269738 | GU320440 | GU384450 |
| *Pseudocercospora musae* | CBS 116634, CBS 121374, UFVMm-46 | GU253775 **KC800750** | EU514438 **KC800777** | **KC800764** | GU269747 | GU320449 | GU384459 |
| *Pseudocercospora nogalesii* | CBS 115022 | — | — | — | GU269752 | GU320454 | GU384463 |
| *Pseudocercospora norchiensis* | CBS 120738 | — | — | — | GU269753 | GU320455 | GU384464 |
| *Pseudocercospora ocimi-basilici* | CPC 10283 | — | — | — | GU269754 | GU320456 | GU384465 |
| *Pseudocercospora ocimicola* | CPC 10283 | GU214678 | — | — | — | — | — |
| *Pseudocercospora paraguayensis* | CPC 1458, CBS 111286 | GU214479 | — | — | DQ267602 | DQ147606 | DQ211680 |
| *Pseudocercospora pini-densiflorae* | MUCC 534 | GU253785 | — | — | GU269760 | GU320461 | GU384471 |
| *Pseudocercospora profusa* | CPC 10042 | — | — | — | GU269787 | GU320488 | GU384497 |
| *Pseudocercospora pseudostigmina-platani* | CPC 11726 | — | — | — | GU269857 | GU320560 | GU384568 |
| *Pseudocercospora punctata* | CPC 10532, CPC 14734 | GQ852645 | — | — | GU269765 | GU320468 | GU384477 |
| **Table S1.** Continued |  |  |  |  |  |  |  |
| **Species** | **Strain** | **LSU** | **mtSSU** | **MCM7** | **ITS** | **Act** | **Tef1** |
| *Pseudocercospora purpurea* | CBS 114163 | — | — | — | GU269783 | GU320486 | GU384494 |
| *Pseudocercospora sambucigena* | CPC 10292 | GU253809 | — | — | GU269788 | GU320489 | GU384498 |
| *Pseudocercospora sordida* | MUCC 913 | — | — | — | GU269777 | GU320480 | GU384488 |
| *Pseudocercospora subtorulosa* | CBS 117230 | — | — | — | GU269816 | GU320518 | GU384528 |
| *Pseudocercospora udagawana* | CPC 10799 | — | — | — | GU269824 | GU320527 | GU384537 |
| *Pseudocercospora vitis* | CPC 11595 | GU214483 | — | — | GU269829 | GU320533 | GU384541 |
| *Pyrenophora phaeocomes* | AFTOL-ID 283 | — | FJ190591 | — | — | — | — |
| *Pyrenophora tritici-repentis* | Pt-1C-BFP | — | — | Genome | — | — | — |
| *Quintaria submersa* | CBS 115553 | GU301866 | — | — | — | — | — |
| *Ramularia coleosporii* | CPC 11516 | GU214692 | — | — | — | — | — |
| *Ramularia endophylla* | CBS 113265, AFTOL-ID 942 | DQ470968 | **KC800779** | JN673014 | — | — | — |
| *Ramularia pratensis var. pratensis* | CPC 11294 | EU019284 | — | — | — | — | — |
| *Ramularia uredinicola* | CPC 10813 | GU214694 | — | — | — | — | — |
| *Ramulispora sorghi* | CBS 110578, CBS 110579 | GQ852653 GQ852654 | — | — | — | — | — |
| *Schizothyrium pomi* | CBS 486.50, CBS 406.61 | EF134948 EF134949 | — | — | — | — | — |
| *Scolecostigmina mangiferae* | CBS 125467, CPC 17352 | GU253877 GU253878 | — | — | — | — | — |
| *Septoria azaleae* | CBS 352.49 | — | DQ028511 | — | — | — | — |
| *Septoria betulae* | bpop01.01 | — | DQ028543 | — | — | — | — |
| *Septoria leucanthemi* | CBS 109090 | GU214492 | — | — | — | — | — |
| *Septoria musiva* | m04.01d, SO2202 | — | DQ028562 | Genome | — | — | — |
| *Septoria populicola* | p01.02b, p02.02b | — | DQ028553 | Genome | — | — | — |
| *Septoria provencialis* | CBS 118910 | — | — | JX142477 | — | — | — |
| **Table S1.** Continued |  |  |  |  |  |  |  |
| **Species** | **Strain** | **LSU** | **mtSSU** | **MCM7** | **ITS** | **Act** | **Tef1** |
| *Septoria ribis* | rib01.02 | — | DQ028512 | — | — | — | — |
| *Septoria rosae* | CPC 4302 | GU214497 | — | — | — | — | — |
| *Septoria rubi* | CPC 12331, rub03.03 | GU253875 | DQ028514 | — | — | — | — |
| *Septoria senecionis* | CBS 102366 | GU214498 | — | — | — | — | — |
| *Setosphaeria turcica* | Et28A | — | — | Genome | — | — | — |
| *Stylodothis puccinioides* | CBS 193.58 | — | AF346428 | — | — | — | — |
| *Teratosphaeria associata* | CBS 112224 | — | — | JN673021 | — | — | — |
| *Teratosphaeria destructans* | CBS 111369 | EU019287 | — | — | — | — | — |
| *Teratosphaeria fibrillosa* | CBS 121707 | GU323213 | — | **KC800765** | — | — | — |
| *Teratosphaeria stellenboschiana* | CBS 124989 | GQ852715 | — | — | — | — | — |
| *Teratosphaeria toledana* | CBS 115513 | FJ493225 | — | — | — | — | — |
| *Trochophora fasciculata* | CPC 10282 | FJ839668 | — | — | — | — | — |
| *Trochophora simplex* | CBS 124744 | GU253880 | — | — | — | — | — |
| *Venturia chlorospora* | Kruys 502 (UPS) | DQ384101 | DQ384084 | — | — | — | — |
| *Verrucisporota proteacearum* | CBS 116003 | FJ839671 | — | — | — | — | — |
| *Zasmidium anthuriicola* | CBS 118742 | FJ839662 | — | — | — | — | — |
| *Zymoseptoria tritici* | CBS 100335, CBS 110744, 687, IPO373 | EU019297 EU019298 | DQ028492 | Genome | — | — | — |

^1^ATCC: American type culture collection, Virginia, USA; CBS: CBS-KNAW Fungal Biodiversity Centre, Utrecht, The Netherlands; CIRAD: Centre de Coopération Internationale en Recherche Agronomique pour le Développement, UMR-BGPI, Montpellier, France; CMW: Culture Collection of the Forestry and Agricultural Biotechnology Institute (FABI) of the University of Pretoria, Pretoria, South Africa; CPC: Culture collection of Pedro Crous, housed at CBS; MUCC: Culture Collection, Laboratory of Plant Pathology, Mie University, Tsu, Mie Prefecture, Japan. AFTOL: Assembling the Fungal Tree of Life (AFTOL) project.

^2^Some sequence for this locus were obtained from: <http://genome.jgi.doe.gov/programs/fungi/index.js>
